# MiR-199a/b-5p inhibits hepatocellular carcinoma progression by post-transcriptionally suppressing ROCK1

**DOI:** 10.18632/oncotarget.18052

**Published:** 2017-05-22

**Authors:** Yangyang Zhan, NanXin Zheng, Fei Teng, Leilei Bao, Fang Liu, Mingjian Zhang, Meng Guo, Wenyuan Guo, Guoshan Ding, Quanxing Wang

**Affiliations:** ^1^ Institute of Immunology and National Key Laboratory of Medical Immunology, Second Military Medical University, Shanghai 200433, China; ^2^ Department of Liver Surgery and Organ Transplantation, Changzheng Hospital, Second Military Medical University, Shanghai 200003, China; ^3^ Department of Pharmacy, No. 411 Hospital of PLA, Shanghai 200080, China

**Keywords:** miRNA, hepatocellular carcinoma, miR-199a/b-5p, metastasis, ROCK1

## Abstract

In this study, we explored the actions of miR-199a/b-5p during hepatocellular carcinoma (HCC) progression and its potential target genes. Through heatmap miRNA expression analysis of 15 matched HCC tumor and adjacent non-tumor liver tissues from the TCGA database, we detected 19 mRNAs that were upregulated and 13 that were downregulated specifically in HCC. Among these, miR-199 family members were downregulated in HCC tumors and cell lines, as compared to controls. Low miR-199a/b-5p expression was also associated with poor overall survival of HCC patients. miR-199a/b-5p overexpression in HCC cell lines inhibited cell proliferation, migration and invasion, both *in vitro* and *in vivo*. In addition, miR199-a/b-5p post-transcriptionally suppressed Rho-associated coiled-coil kinase 1 (ROCK1). This in turn led to inhibition of ROCK1/MLC and PI3K/Akt signaling, which is necessary for HCC proliferation and metastasis. These results indicate that miR-199a/b acts as tumor suppressors in HCC and represent promising therapeutic targets.

## INTRODUCTION

Hepatocellular carcinoma (HCC) is the fifth most common cancer worldwide with a high rate of metastasis and recurrence and ranks third in cancer-related deaths [1, 2]. China alone accounts for more than 50% of HCC cases worldwide [3], while its incidence and mortality rates are rapidly growing in developed countries [4, 5]. However, the molecular mechanisms underlying metastasis and recurrence of HCC are complex and need better understanding, especially to identify novel therapeutic targets for this grave disease.

MicroRNAs (miRNAs) are endogenous, single-stranded, small non-coding RNAs that act as transcriptional or post-transcriptional regulators of gene expression through translational repression or transcript cleavage [6]. Recent evidence suggests that miRNAs can function as tumor suppressors by negatively regulating oncogenes or as tumor promoters by downregulating tumor suppressor genes [7]. Thus, miRNAs are considered as potential targets for cancer therapy. Among the miRNAs, miR-199 is of great interest for cancer therapies because it’s associated with various tumors including prostate cancer [8], breast cancer [9], medulloblastoma [10], and osteosarcoma [11]. However, the mechanistic details that are therapeutically relevant regarding the role of miR-199 in HCC are not known. Previously, we showed that miR-199a/b-3p was the third most abundant miRNA in human liver tissues and was downregulated in HCC tumors [12]. Therefore, in this study, we explored the tumor suppressor role of miR-199a/b-5p in HCC and its potential targets by analyzing HCC patient data and cell lines.

## RESULTS

### Downregulation of miR-199 correlates with poor survival of HCC patients

**Figure 1:**
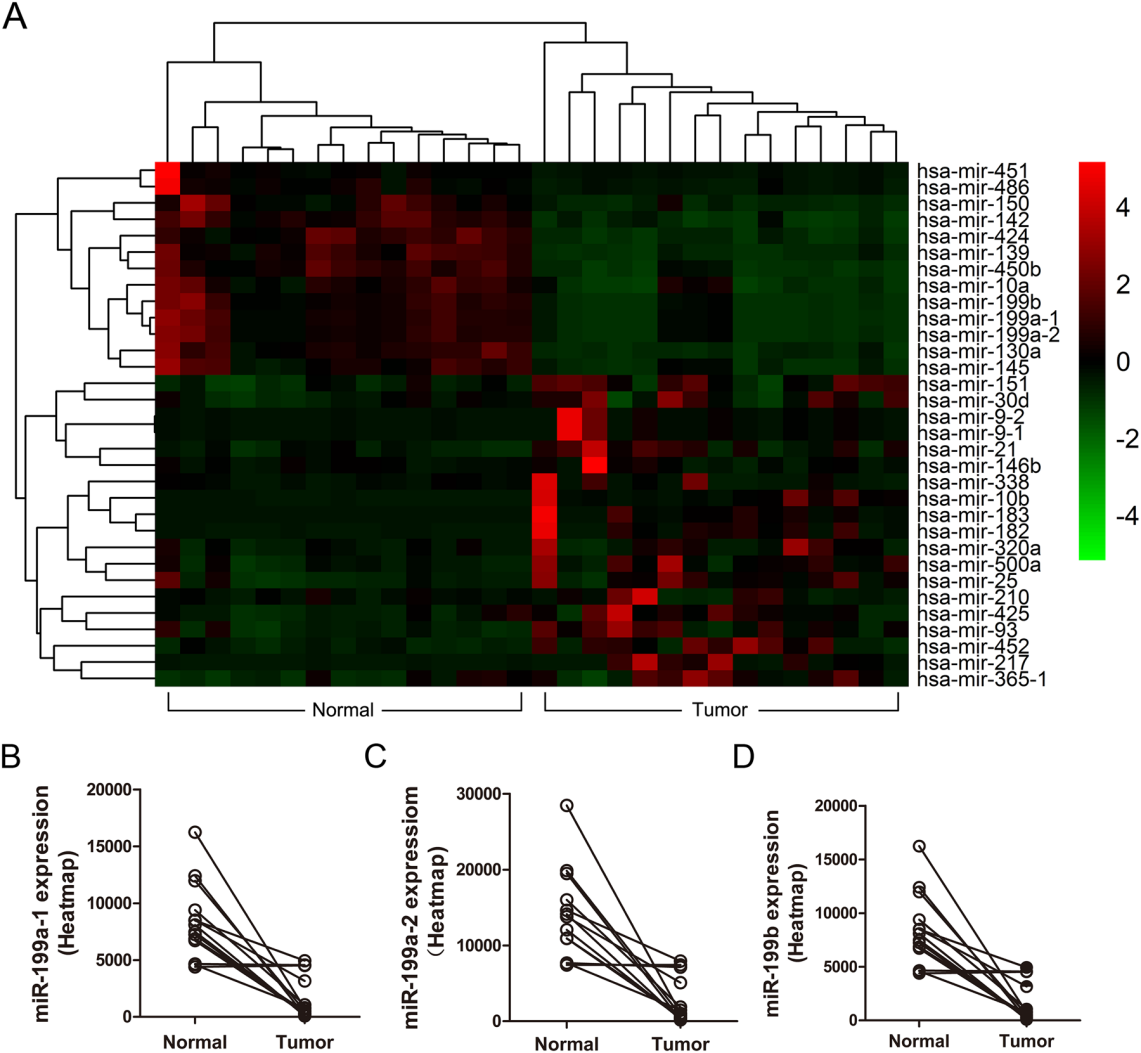
Heatmap analysis of miRNA expression in human HCC patient samples **(A)** The heatmap analysis was performed by R software with DESeq package (p <0.05 and log_2_ fold change >1) to illustrate miRNA expression profiles in 15 HCC cases and matched normal liver tissues. **(B-D)** The expression levels of miR-199a-1, miR-199a-2, miR-199b in HCC tumor and normal liver tissues based on the heatmap miRNome profiles for 15 HCC patient samples (tumor and adjacent normal liver tissues).

We first performed heatmap analysis of the miRNA expression profiles in 15 matched HCC tumor and normal liver tissues in the TCGA database. Supplementary Table 1 shows the top miRNAs with >300 average reads and at least one-fold difference. Our data identified 19 upregulated and 13 downregulated miRNAs in HCC tissues compared with matched normal liver tissues (Figure 1A). Among these, miR-199a-1, miR-199a-2, miR-199b were highly downregulated in the HCC tissues (Figure 1B-1D).

**Figure 2:**
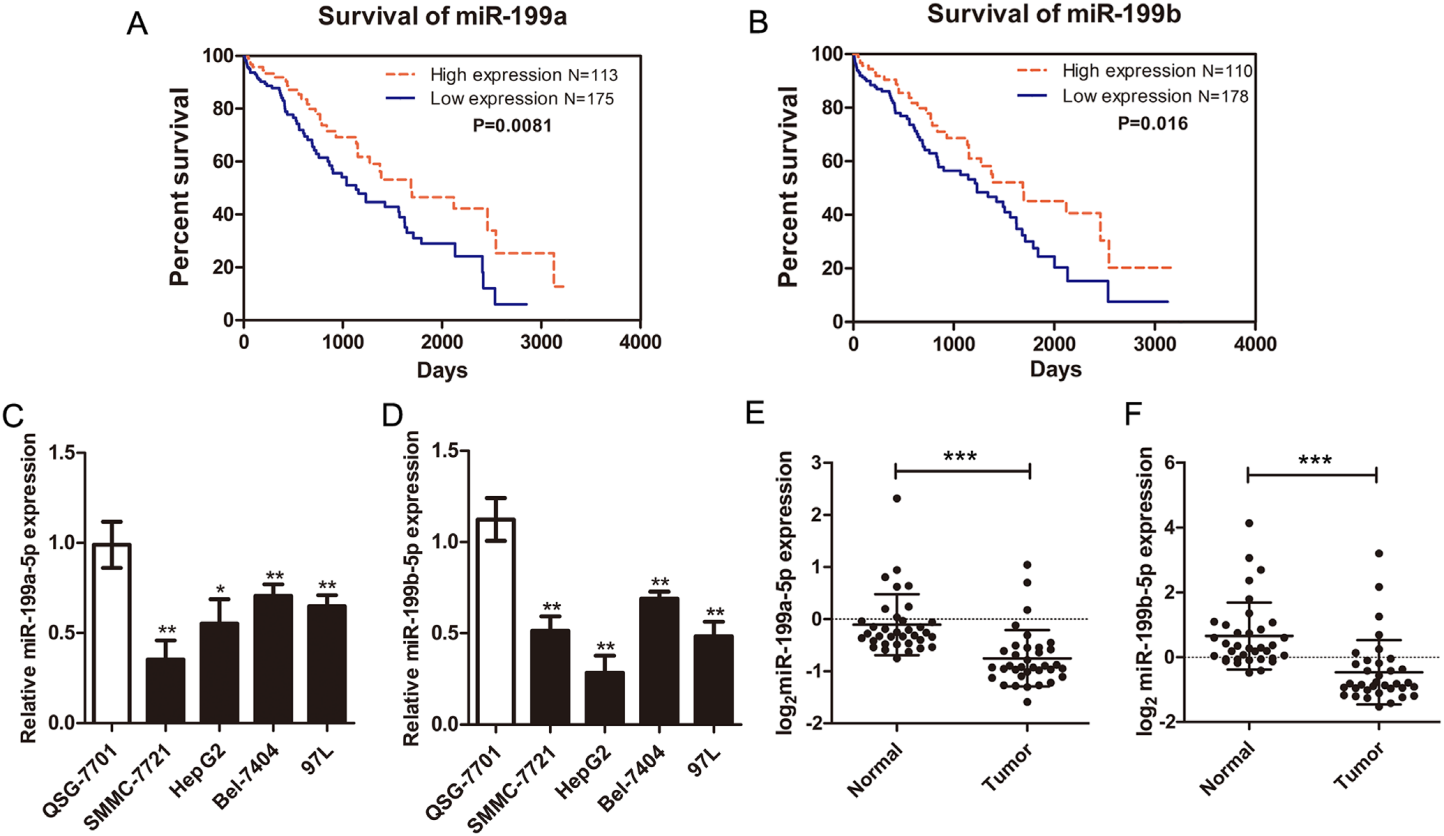
Aberrant miR-199a/b-5p expression in HCC tumors and cell lines **(A-B)** Kaplan-Meier survival curves showing relationship between high or low miR-199a/b expression levels (the median value was chosen as the cutoff point) with overall survival of HCC patients (288 clinical samples from TCGA project). **(C-D)** The relative expression of miR-199a/b-5p in immortalized normal hepatic cell line, QSG-7701, and HCC cell lines, SMMC-7721, HepG2, Bel-7404, and 97L was detected by qRT-PCR. The expression of miR-199a/b-5p was normalized to U6 in each sample. **(E-F)** The qRT-PCR analysis of miR-199a/b-5p expression in 35 matched human HCC and corresponding adjacent normal tissues. The results were expressed as mean ± SD (*p<0.05, **p<0.01, ***p<0.001).

We then evaluated the association between miR-199a/b expression levels with survival of HCC patients from the TCGA project. The HCC patients were divided into high or low miR-199a/b groups and subjected to Kaplan-Meier analysis. Our data showed that patients with high expression of miR-199a/b showed increased overall survival compared to those with low miR-199a/b levels (Figure 2A, 2B). These results suggested that miR-199 played a tumor suppressor role in HCC.

### MiR-199a/b-5p is downregulated in HCC tumor tissues and cell lines

Next, we analyzed the miR-199a/b-5p levels in HCC cell lines, SMMC-7721, HepG2, Bel-4404, and 97L in comparison to liver immortalized QSG-7701 cells. qRT-PCR analysis showed that both miR-199a-5p and miR-199b-5p were downregulated in the SMMC-7721, HepG2, Bel-4404, and 97L cells compared to QSG-7701 cells (Figure 2C, 2D). To further confirm these results, we analyzed the expression of miR-199a-5p and miR-199b-5p in 35 pairs of snap-frozen primary HCC and their corresponding non-tumor liver specimens. Consistent with the findings in HCC cell lines, the expression miR-199a-5p and miR-199b-5p in HCC tumors was lower than the corresponding non-tumor liver tissues (Figure 2E, 2F). Together, these results revealed that both miR-199a-5p and miR-199b-5p were downregulated in HCC.

### MiR-199a/b-5p overexpression inhibits HCC proliferation and metastasis

**Figure 3:**
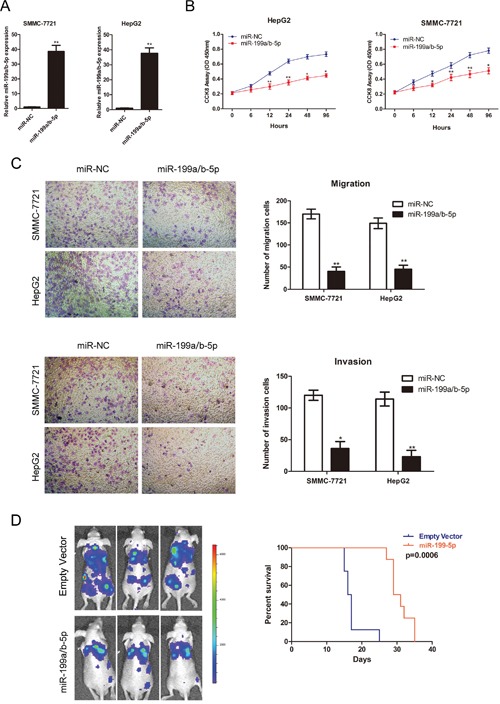
MiR-199a/b-5p overexpression reduces HCC proliferation and metastasis **(A)** The relative expression of miR-199a/b-5p in SMMC-7721 and HepG2 cells transfected with 80nM miR-199a/b-5p mimics (1:1) or control. **(B)** Estimation of cell proliferation in SMMC-7721 and HepG2 cell lines transfected with control or miR-199a/b-5p mimics by cell counting kit-8 (CCK-8) assays. **(C)** Transwell assays determining cell migration and invasion potential of SMMC-7721 and HepG2 cell lines transfected with control or miR-199a/b-5p mimics. **(D)** *In vivo* xenograft tumor nude mice model determining the effects of miR199a/b-5p overexpression on HCC metastasis and mice survival. SMMC-7721 cells with control or stably overexpressed miR-199a/b-5p were injected into the caudal vein of mice and examined 14 days after cells implantation at the *In Vivo* Imaging System (IVIS). Kaplan-Meier survival curves of mice implanted with control or stably overexpressed miR-199a/b-5p SMMC-7721 cells are shown. The results are expressed as mean ± SD (*p<0.05, **p<0.01).

We postulated that downregulation of miR-199a/b-5p in HCC was critical for HCC progression. Therefore, we assessed HCC proliferation, migration and invasion by transfecting miR-199a/b-5p mimics in the HCC cell lines. We demonstrated that transfection of miR-199a/b-5p mimics increased miR-199a/b-5p expression in HCC cells lines (Figure 3A, Supplementary Figure 1A). Consequently, CCK8 assay demonstrated diminished cell proliferation in miR-199a/b-5p transfected cell lines compared to controls (Figure 3B, Supplementary Figure 1B). Also, plate colony formation assays demonstrated that HCC cells transfected with miR-199a/b-5p mimics formed fewer colonies than those transfected with the control miR-NC (Supplementary Figure 2A).

Firstly, we performed wound-healing assays to analyze the role of miR-199a/b-5p in motility of HCC cells. We observed that HCC cells transfected with miR-199a/b-5p mimics showed reduced ability to fill up the scratch wounds compared with the control group at 24h after wounding (Supplementary Figure 2B, 2C). Further, transwell assays to analyze HCC migration and invasion demonstrated that overexpression of miR-199a/b-5p in HCC cells markedly decreased cell migration and invasion compared to the controls (Figure 3C, Supplementary Figure 1C). Next, we stably overexpressed miR-199a/b-5p in SMMC-7721 cells (Supplementary Figure 2D) and injected the cells into caudal vein of immunodeficient nude mice to investigate the role of miR-199a/b-5p in HCC metastasis *in vivo*. As shown in Figure 3D, the overexpression of miR-199a/b-5p repressed metastasis and prolonged mice survival, further confirming that miR-199a/b-5p expression suppressed HCC metastasis.

### MiR-199a/b-5p post-transcriptionally regulates ROCK1

**Figure 4:**
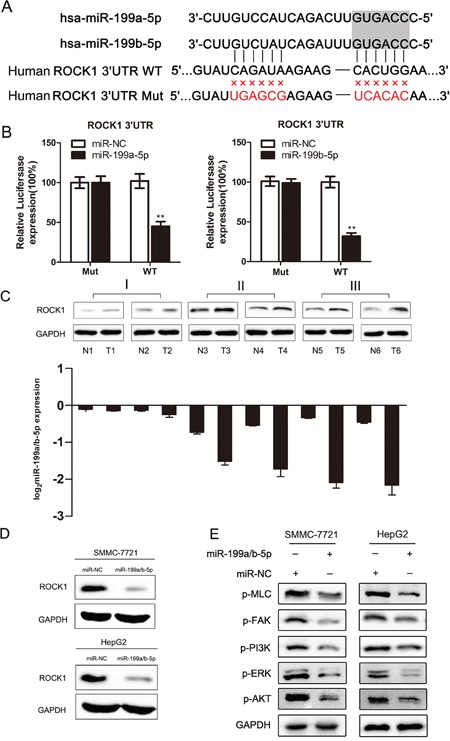
MiR-199a/b-5p post-transcriptionally inhibits ROCK1 **(A)** Targetscan analysis demonstrating that miR-199a/b-5p targets ROCK1 3’UTR sites. The schematic graph shows the putative binding sites of miR-199a/b-5p in the ROCK1 3’UTR and the mutations in the miR-199a/b-5p binding sites. **(B)** Relative luciferase activity in ROCK1 3’UTR wild-type (WT) and ROCK1 3’UTR mutant type (Mut) demonstrating that miR-199a/b-5pmimics downregulate luciferase activity from the wild-type ROCK1 3’UTR, but did not alter luciferase activity from the mutant ROCK1 3’UTR. **(C)** Expression of ROCK1 in representative stage I to stage III HCC tumor samples. The corresponding miR-199a/b-5p expression levels are shown below. **(D)** Western blot analysis of ROCK1 expression in SMMC-7721 and HepG2 transfected with miR-NC and miR-199a/b-5p mimics. **(E)** Western blot analysis of the ROCK1/MLC and PI3K/AKT signaling pathways in SMMC-7721 and HepG2 cell lines transfected with miR-NC or miR-199a/b-5p mimics. The results are expressed as mean ± SD (*p<0.05, **p<0.01).

Next, we searched the TargetScan database to identify miR-199a/b-5p targets that are critical in repressing HCC progression. We identified 621 putative targets through Targetscan database (Supplementary Table 2). Then we subjected these putative targets to pathway enrichment analysis using Kyoto Encyclopedia of Genes and Genomes (KEGG) pathway database (Supplementary Table 3). We consistently found ROCK1 and ARHGEF12 as potential miR199a/b-5p targets (Supplementary Figure 3A). To confirm the database predictions, we performed dual-luciferase reporter assays and observed that miR-199a/b-5p mimics inhibited the luciferase activity of the ROCK1 3’-UTR reporter, but did not affect the luciferase activity of a ROCK1 reporter with mutated miR-199a/b-5p binding sites (Figure 4A, 4B). However, co-transfection of miR-199a/b-5p mimics did not inhibit the luciferase activity of the ARHGEF12 3’-UTR reporter (data not shown) suggesting that it was not a bonafide target of miR199a/b-5p.

Then, we analyzed ROCK1 expression in paired primary HCC and normal liver tissues and found that miR-199a/b-5p negatively correlated with ROCK1 expression and HCC progression (Figure 4C). Furthermore, transfection of miR-199a/b-5p mimics decreased the expression of ROCK1 protein in HCC cell lines but did not affect its mRNA levels (Figure 4D, Supplementary Figure 3B). This suggested that miR-199a/b-5p inhibited ROCK1 translation and thereby suppressed HCC progression.

Since ROCK1 is involved in proliferation, migration, and invasion of cancer cells, we analyzed protein expression and phosphorylation of key signaling molecules associated with ROCK1 [13, 14]. As shown in Figure 4E, we observed decreased p-MLC, p-FAK, p-PI3K, p-ERK, and p-AKT in HCC cells transfected with miR-199a/b-5p mimics compared with the relevant controls.

### ROCK1 knockdown inhibits HCC progression

**Figure 5:**
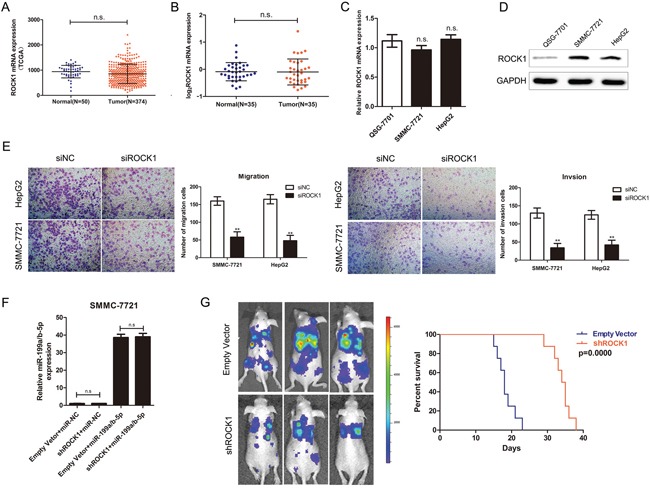
ROCK1 regulates HCC migration, and invasion **(A)** ROCK1 mRNA expression levels in 50 normal liver and 374 HCC tumor samples from the TCGA RNA-Seq database. **(B)** qRT-PCR analysis of ROCK1 mRNA levels in 35 HCC and matched normal liver samples. **(C)** qRT-PCR analysis of ROCK1 mRNA levels in immortalized normal hepatic cell line, QSG-7701 and HCC cell lines, SMMC-7721 and HepG2. **(D)** Western blot analysis of ROCK1 protein in immortalized normal hepatic cell line, QSG-7701 and HCC cell lines, SMMC-7721 and HepG2. **(E)** Transwell assays showing cell migration and invasion potential of control and ROCK1 siRNA transfected SMMC-7721 and HepG2 cell lines. **(F)** qRT-PCR analysis of miR-199a/b-5p expression in ROCK1 knockdown SMMC-7721 cells transfected with miR-NC or miR-199a/b-5p. **(G)** *In vivo* xenograft tumor nude mice model determining the effects of ROCK1 knockdown on HCC metastasis. Control and ROCK1 siRNA transfected SMMC-7721 cells were injected into the caudal vein of mice and examined after 14 days at the *In Vivo* Imaging System (IVIS). Kaplan-Meier survival curves of mice implanted with control and ROCK1 siRNA transfected SMMC-7721 cells are shown. The results are expressed as mean ± SD (*p<0.05, **p<0.01, n.s means no significance).

Next, we examined the role of ROCK1 in HCC progression by analyzing the patient samples from the TCGA database. We observed no significant differences in the ROCK1 mRNA levels between primary tumor and non-tumor liver samples (Figure 5A, 5B). Consistently, we observed increased ROCK1 protein in HCC cell lines and primary tumor samples compared to normal liver cell line and tissue samples (Figure 4C, Figure 5D), but the mRNA levels remained unchanged (Figure 5C). Next, we downregulated ROCK1 expression by transfecting HCC cell lines with siRNAs against ROCK1 and found that one of three siRNAs significantly inhibited ROCK1 expression. The transfection efficiency of the 3 siRNAs in the SMMC-7721 and HepG2 cell lines were analyzed by qRT-PCR (Supplementary Figure 4A). Then, we observed that ROCK1 knockdown reduced the ability to fill up the scratch wounds at 24h after wounding (Supplementary Figure 4C). Also, we analyzed the effect of ROCK1 knockdown on the migration and invasion properties of the SMMC-7721 and HepG2 cell lines by Transwell assays. Our data demonstrated that knockdown of ROCK1 inhibited HCC cell migration and invasion compared to the controls (Figure 5E).

Furthermore, we transfected miR-NC or miR-199a/b-5p into ROCK1 knockdown and control SMMC-7721 cells (Supplementary Figure 4B), and observed that knockdown of ROCK1 has no effect on miR-199a/b-5p expression compared to control cells (Figure 5F). The results of transwell assays showed that overexpression miR-199a/b-5p in ROCK1 knockdown SMMC-7721 cells has no remarkable difference on migration and invasion compared to cells transfected with miR-NC (Supplementary Figure 4D). These results demonstrated that ROCK1 is the main target of miR-199a/b-5p in inhibiting HCC migration and invasion. Then, we xenografted ROCK1 knockdown and control SMMC-7721 cells in the nude mice, and observed that knockdown of ROCK1 suppressed metastasis of tumor cells in the lungs and liver and consequently enhanced mice survival times (Figure 5G). These findings further demonstrated that ROCK1 promoted HCC metastasis.

## DISCUSSION

In recent years, miRNAs have emerged as important regulators of tumorigenesis [15, 16]. In this study, heatmap analysis of miRNA expression in patient samples demonstrated that miR-199a/b-5p was a HCC-specific miRNA. The following *in vitro* and *in vivo* assays demonstrated that miR-199a/b-5p was a critical tumor suppressor in HCC that post-transcriptionally inhibited ROCK1 expression and thereby inhibited ROCK1/MLC and PI3K/AKT pathways that are necessary for HCC progression.

Heatmap analysis of the HCC patient miRNA expression profile in the TCGA datasets identified 32 HCC-specific miRNAs. These included previously reported HCC-specific miRNAs like miR-21, miR-139, miR-9, and miR-10a that corroborated our findings [17–20]. In addition, we found several novel HCC-specific miRNAs including miR-450b, miR-146b, miR-30d, miR-500a and further studies are required to decipher their role in HCC. Since miRNAs repress expression of their target genes, a minimum threshold expression of miRNAs is necessary to exert their function [21, 22]. Thus, miRNAs expressed highly are considered more important. Among these HCC-specific miRNAs, miR-199-a/b was specifically abundant in normal liver tissues and significantly decreased in HCC tissue samples. In addition, low miR-199a/b levels correlated with the survival of HCC patients suggesting its critical role in HCC progression.

The miR-199 members have emerged as important tumor markers. The downregulation of miR-199a/b was observed in non-small cell lung cancer [23], pancreatic tumors [24] and testicular germ cell tumors [25], whereas its upregulation was demonstrated in gastric cancer [26], Marek’s disease-induced T-cell lymphoma [27] and uveal melanoma [28]. These results suggested diverse functions for miR-199 members in different cancers. Recent studies suggested that miR-199 was necessary for progression from chronic hepatitis to liver cirrhosis and finally to HCC. It was also reported that miR-199 modulated hepatitis B viral replication [29]. Also, miR-199a correlated with progression of liver fibrosis [30]. Additionally, miR-199 members are recognized as putative targets for diagnosis and treatment of HCC [31, 32].

The miRNAs are involved in repressing the expression of their targets by complementary base-pairing [33]. Hence, it was important to identify the exact functions of each member of the miR-199 family in HCC. Generally, a mature miRNA has two different sequences called 3p and 5p, which are generated from 3’ and 5p’ regions of pre-miRNA, respectively [34]. Previously, we reported that miR-199a/b-3p was downregulated in HCC and associated with HCC proliferation [12]. In the present study, we found that miRNA-199a/b-5p was significantly decreased in both HCC tumors and cell lines. Moreover, overexpression of miR-199a/b-5p suppressed SMMC-7721 and HepG2 cell proliferation, migration, and invasion. Further, we demonstrated that overexpression of miR-199a/b-5p in HCC cells repressed their ability to metastasize *in vivo* and hence prolonged survival of the xenografted nude mice. These data showed that miR-199a/b-5p functioned as a tumor suppressor of HCC.

The tumor suppressor or oncogenic role of miRNAs depends on the role of their specific target genes in cancer [35]. Recent studies have revealed that several potential targets of miR-199 are involved in carcinogenesis and metastasis. Upregulation of miR-199a/b inhibits cell proliferation, migration and invasion in non-small cell lung cancer by suppressing Axl expression [23]. Overexpression of miR-199a suppresses renal cancer cell proliferation and survival by downregulating GSK-3β expression [36]. Also, downregulation of miR-199a resulted in high expression of ERBB2 and ERBB3 in ovarian cancer cells and promoted cancer progression [37]. Although several targets of miR-199 have been identified, their relevance in promoting cancer is not fully understood. Therefore, we performed Targetscan analysis to identify new targets of miRNA-199a/b-5p in HCC and identified many putative target genes of miR-199a/b-5p. Further, using KEGG pathway enrichment analysis, we observed that ROCK1 and ARHGEF12 were found in the 4 most enriched cell signaling pathways. Dual-luciferase reporter assays demonstrated that ROCK1 was the bonafide target gene of miR-199a/-5p relevant for HCC. Further experiments showed that miR-199a/b-5p suppressed the expression of ROCK1 at the translational level.

ROCK is a Ser/Thr protein kinase and a major downstream effector of Ras homolog family member A (RhoA) signaling [38, 39]. RhoA/Rock signaling pathway affects many cellular processes, including mitochondrial fission, signal transduction, and cytoskeletal organization [40, 41]. ROCK1, the leading member of ROCK family is an important regulator of carcinogenesis [41–43]. Increased expression of ROCK1 is reported in several human cancers and correlated with poor survival [42]. ROCK1 enhances cancer cell motility and invasiveness by modulating reorganization of the actin cytoskeleton [41, 43]. Our data showed that mRNA expression levels of ROCK1 were similar in primary tumor and matched non-tumor liver samples as well as in HCC cell lines. This was contrary to a previous study that reported high ROCK1 mRNA and protein levels in breast cancer tissues compared with to the paired normal tissues [42]. Our further experiments established that ROCK1 protein levels were enhanced in both HCC cell lines and tissue samples compared to normal controls. We also found that knockdown of ROCK1 has no effect on miR-199a/b-5p expression and overexpression of miR-199a/b-5p in ROCK1 knockdown cells has no effect on cell migration and invasion compared to control cells. These findings indicated post transcriptional regulation ROCK1 by miR-199a/b-5p.

In a previous study, inhibitor of ROCK1 suppressed the malignant phenotype of breast cancer by attenuating the ability of the cancer cells to migrate and invade through ROCK/MLC signaling [14]. In this study, we demonstrated that knockdown of ROCK1 reduced *in vivo* and *in vitro* HCC metastasis, and prolonged survival of xenografted mice. The role of the ROCK/MLC pathway is to regulate actin dynamics [43, 44]. Our results showed that inhibition of ROCK1 by miR-199a/b-5p decreased ROCK1/MLC signaling, thereby suppressing cell migration and invasion. Stabilized focal adhesion kinase (FAK) is crucial for directional cell migration [45]. Previously, it was shown that PI3K/AKT and Ras/ERK1/2 were downstream targets of FAK/Src [46]. In our study, downregulation of ROCK1 resulted in decreased p-FAK, p-PI3K, p-ERK and p-AKT. Thus, we showed that downregulation of ROCK1 by miR-199a/b-5p lowered FAK/Src activity that subsequently reduced PI3K/AKT signaling, thereby suppressing HCC metastasis.

In conclusion, our study demonstrated that miR-199a/b-5p acted as a tumor suppressor by post-transcriptionally suppressing ROCK1 that resulted in diminished ROCK1/MLC and PI3K/AKT signaling, thereby inhibiting HCC metastasis. Hence, our study revealed that miR-199a/b-5p is a potential therapeutic target for HCC.

## MATERIALS AND METHODS

### The cancer genome atlas (TCGA) datasets

The TCGA contains miRNA array, RNA-Seq and clinical datasets for various cancers. We obtained fifteen paired samples from a primary HCC miRNA array in the TCGA for heatmap analysis. We also used 288 HCC miRNA array datasets and related clinical data from TCGA project to analyze overall survival. Further, we obtained 374 HCC and 50 normal liver tissue mRNA-Seq datasets from TCGA to analyze ROCK1 mRNA expression.

### HCC patient samples

We obtained 35 pairs of HCC and matched adjacent liver tissue samples from the Department of Liver Surgery and Organ Transplantation, Changzheng Hospital, Second Military Medical University (Shanghai, China). The patients had no history of chemotherapy or radiotherapy before surgical resection. The clinical stages of HCC tumors were determined according to the TNM classification system of the International Union Against Cancer (7th edition). The research protocol was approved by the Ethics Committee of Second Military Medical University, and informed written consent was obtained from all patients according to the Declaration of Helsinki and its amendments.

### Cell culture and transfection

Human HCC cell lines SMMC-7721, HepG2, Bel-7404, 97L, human hepatocyte QSG-7701 cells and HEK293 cells were obtained from American Type Culture Collection (ATCC, USA). The SMMC-7721, HepG2, QSG-7701 and HEK293 cells were cultured in RPMI-1640 (Invitrogen, USA) with 10% fetal bovine serum (BioWest, France).

For transfection experiments, 2.5×10^5^ HCC cells were seeded in a 6-well plate and incubated overnight before transfection with miRNA mimics or siRNAs. The miR-199a-5p and miR-199a-5p mimics were synthesized by GenePharma (China) and mixed in 1:1 ratio to overexpress miR-199a/b-5p. Three siRNAs (GenePharma, China) targeting human ROCK1 mRNA (siRNA1: CTTCCTCGAACGCTTTCTAC-3’;siRNA2:5’-ACACTCTACCACTTTCCTGCA-3’; siRNA3: 5’-ATGAGCTTCAGATGCAGTTCCA-3’) and a non-specific siRNA were also synthesized. Lipofectamine2000 (Invitrogen, USA) was used for RNA transfections according to manufacturer’s recommendations.

### Dual luciferase reporter assays

Dual-luciferase reporter assay (Promega, Madison, WI) was performed according to the manufacturer’s instructions. ROCK1 or ARGEH12 3’UTR sequences were PCR amplified and cloned into the pGL3 vector. HEK293 cells were plated at a density of 1×10^3^ cells/well in a 96 well plate and transfected with 80ng of luciferase reporter and 40ng of pRL-TK-Renilla-luciferase plasmids with 20nM indicated RNAs. The firefly luciferase activity was normalized with renilla luciferase activity to obtain transfection efficacy.

### Quantitative RT-PCR analysis

Total RNA was extracted from cell lines and patient samples using Trizol (Invitrogen) according to the manufacturer’s instructions. RNA was reverse transcribed and detected by real-time PCR as described previously [12]. For reverse transcription of miRNA-199a/b-5p, the primer was 5’– CTCAACTGGTGTCGTGGAGTCGGCAATTCAGTTGAGGAACAGGT–3’ and for real time PCR, the primers for miR-199a/b-5p were 5’- ACACTCCAGCTGGGAGTGTTCAGACTAC-3’ (forward) and 5’-TGGTGTCGTGGAGTCG -3’ (reverse). The qRT-PCR data was normalized to U6 expression in each sample. For analysis of ROCK1 mRNA expression, the primers were 5’- AACATGCTGCTGGATAAATCTGG-3’ (forward) and 5’-TGTATCACATCGTACCATGCCT-3’ (reverse). Data were normalized to GAPDH expression in each sample.

### Cell proliferation assay

Cell proliferation assays were performed with the CCK-8 Kit (Dojindo, Shanghai, China) according to the manufacturer’s instructions. HCC cells transfected with miR-199a/b-5p or miR-NC mimics were seeded in 96-well plates (200μl/well) at a density of 1×10^4^ cells/well. Then, the transfected cells were incubated with CCK-8 (20μl/well) at 37°C for an additional 1.5h. Optical density (OD) values were determined at 490nm using an automated ELISA plate reader. The experiment was performed for a 96h timeline with a time point every 6h.

### Cell migration and transwell assay

Migration assay was carried out in Transwell chambers with 8.0μm pore size (BD Biosciences, USA). HCC cells transfected with miR-199a/b-5p or miR-NC mimics (2.5×10^4^/well) were seeded in serum-free medium in the upper chamber and medium with 10% FBS was added to the lower chamber. After incubation at 37°C for 24h, the cells on the lower surface of the membrane were fixed and stained with 0.01% crystal violet. The migrated cells were counted for all samples under a microscope at 200× magnification.

For invasion assay, the upper chamber was pre-coated with 1mg/ml matrigel (BD Biosciences) and the assay was s performed as described for the migration assay above.

### Western blot analysis

For protein extraction, cells and tissues were lysed with M- and T-PER protein extraction reagent (Thermo, USA), respectively, supplemented with complete protease inhibitor cocktail (Roche, USA). The total protein in the samples was determined by the BCA assay (Thermo, USA). Equal amounts of total protein from various samples was separated by SDS-PAGE and transferred onto nitrocellulose membranes (Millipore, USA). The blots were probed with the following antibodies: ROCK1 (#4035); p-MLC (#3674); p-FAK (#8556); p-PI3K (#13857); p-ERK (#4370); p-AKT (#9614); GAPDH (#5174) (CST, USA). Then, after washes, the blots were incubated with secondary rabbit antibody. Then the blots were detected by chemiluminescence detection (Tanon 4200, China) and the protein bands were quantified by ImageJ 1.49v (Wayne Rasband National Institutes of Health, USA).

### Generation of stable miR-199a/b-5p overexpressing and ROCK1 knockdown cell lines

Recombinant lentiviruses containing pre-hsa-miR-199a/b-5p or the control were purchased from GeneChem (China). SMMC-7721 cells were transfected with 2×10^6^ transducing units of miR-199a/b-5p overexpression lentiviruses and stable tranfectants were selected with 2μg/ml puromycin (GeneChem) for three weeks. Similarly, recombinant lentiviruses with shRNA targeting human ROCK1 mRNA or the control were purchased from GeneChem (China). SMMC-7721 cells were transfected with 2×10^6^ transducing units of shRNA-ROCK1 lentiviruses and selected with 2μg/ml puromycin (GeneChem) for three weeks. The stable cell lines were verified using qRT- PCR.

### Xenograft mouse model to determine *in vivo* tumorigenicity

The animal experiments were approved by the Scientific Investigation Board of Second Military Medical University and performed in accordance with the guidelines of National Institute of Health Guidance for the Care and Use of Laboratory Animals. Six-week-old male BALB/c nude mouse were purchased from Sippr-BK laboratory animal Co. Ltd (China). To establish nude mice metastasis model, 1×10^6^ SMMC-7721 cells (transfected with miR199a/b-5p or control mimics and ROCK1 or control shRNA) were injected into the caudal vein of mice. At 14 days after injection of SMMC-7721 cells, the metastases was monitored using the IVIS@Lumina II system (Caliper Life Sciences, MA), 15min after 3mg luciferin (GeneChem, China) was intraperitoneally injected.

### Statistical analysis

GraphPad Prism software 6.0 (GraphPad Software, San Diego, CA, U.S.) was used for statistical analysis and figure preparation. Experimental data were expressed as mean±standard deviation (SD) or as percentages. A Student’s t-test was used to compare two groups of data. Differences were deemed statistically significant at *p < 0.05, **p< 0.01, ***p< 0.001.

## SUPPLEMENTARY MATERIALS AND METHODS FIGURES AND TABLES




