# Supplementary material for: MiR-199a/b-5p inhibits hepatocellular carcinoma progression by post-transcriptionally suppressing ROCK1
Source: Oncotarget. 2017 May 22;8(40):67169–80. doi: 10.18632/oncotarget.18052 (PMC5620164; doi:10.18632/oncotarget.18052)
Supplement: Supplementary file 1 [file oncotarget-08-67169-s001.pdf]

MiR-199a/b-5p inhibits hepatocellular carcinoma progression by post-transcriptionally suppressing ROCK1

SUPPLEMENTARY MATERIALS AND METHODS

Colony formation assay

For colony formation assays, 500 HCC cells transfected with miR-199a/b-5p or miR-NC mimics were plated in 35 mm culture dish and incubated at 37°C for 2 weeks. Then, the cells were washed twice with phosphate-buffered saline (PBS), fixed in 2ml 4% paraformaldehyde for 30 min and further stained with 1ml 0.1% crystal violet for 20 min followed by 3 washes with ddH$_2$O. The plates were then photographed and analyzed.

Wound healing assay

For wound healing assays, HCC cells were cultured to form a monolayer in 6-well plates. The monolayer was manually scratched with a pipette tip to form a wound and observed under an inverted microscope at 0 and 24h to analyze the extent of healing.

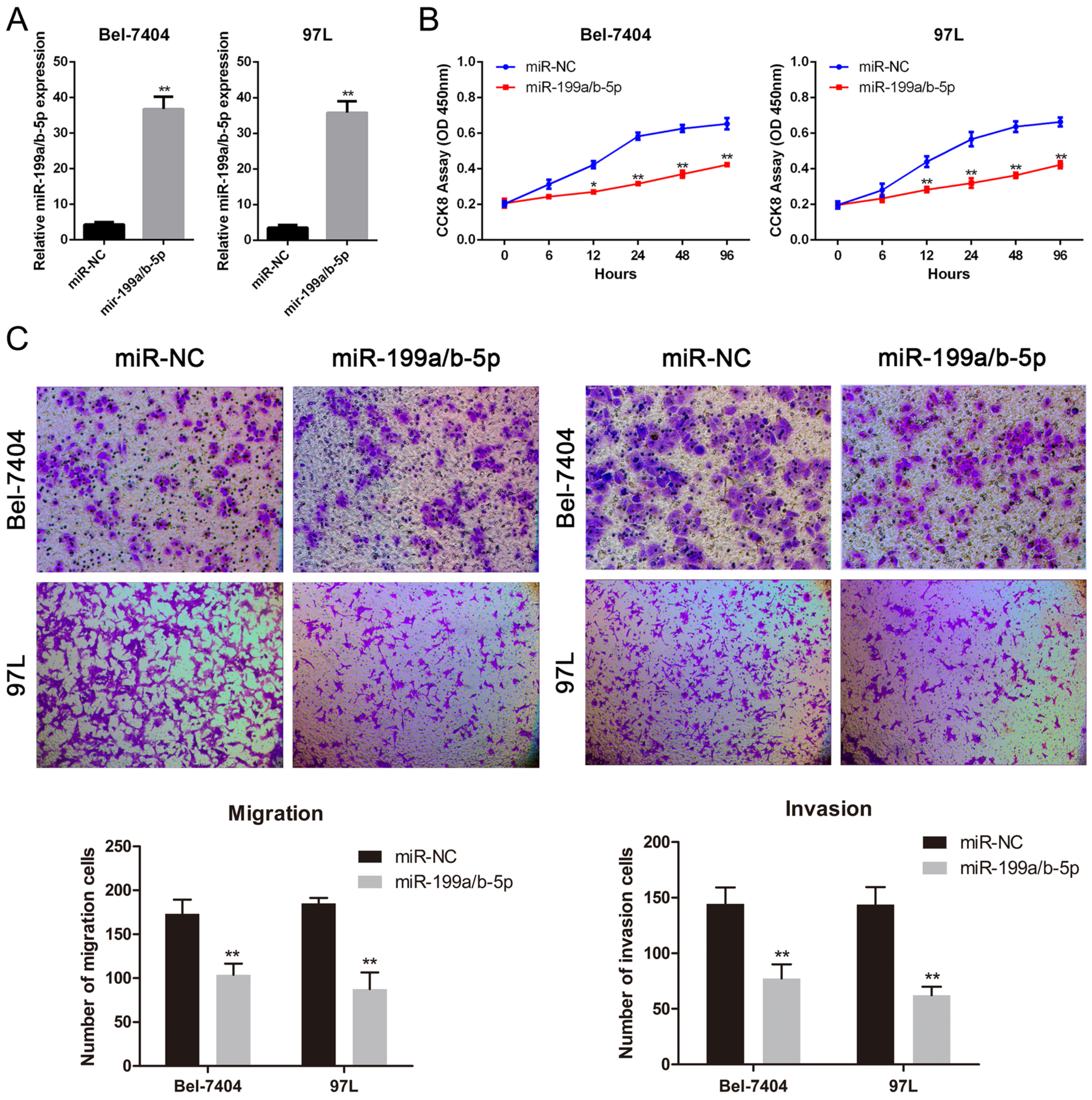

Supplementary Figure 1: Related to Figure 3. (A) The relative expression of miR-199a/b-5p in Bel-7404 and 97L cells transfected with 80nM miR-199a/b-5p mimics (1:1) or control. **(B)** Estimation of cell proliferation in Bel-7404 and 97L cells transfected with control or miR-199a/b-5p mimics by cell counting kit-8 (CCK-8) assays. **(C)** Transwell assays determining cell migration and invasion potential of Bel-7404 and 97L cell lines transfected with control or miR-199a/b-5p mimics. The results were expressed as mean ± SD (*$p<0.05$, **$p<0.01$).

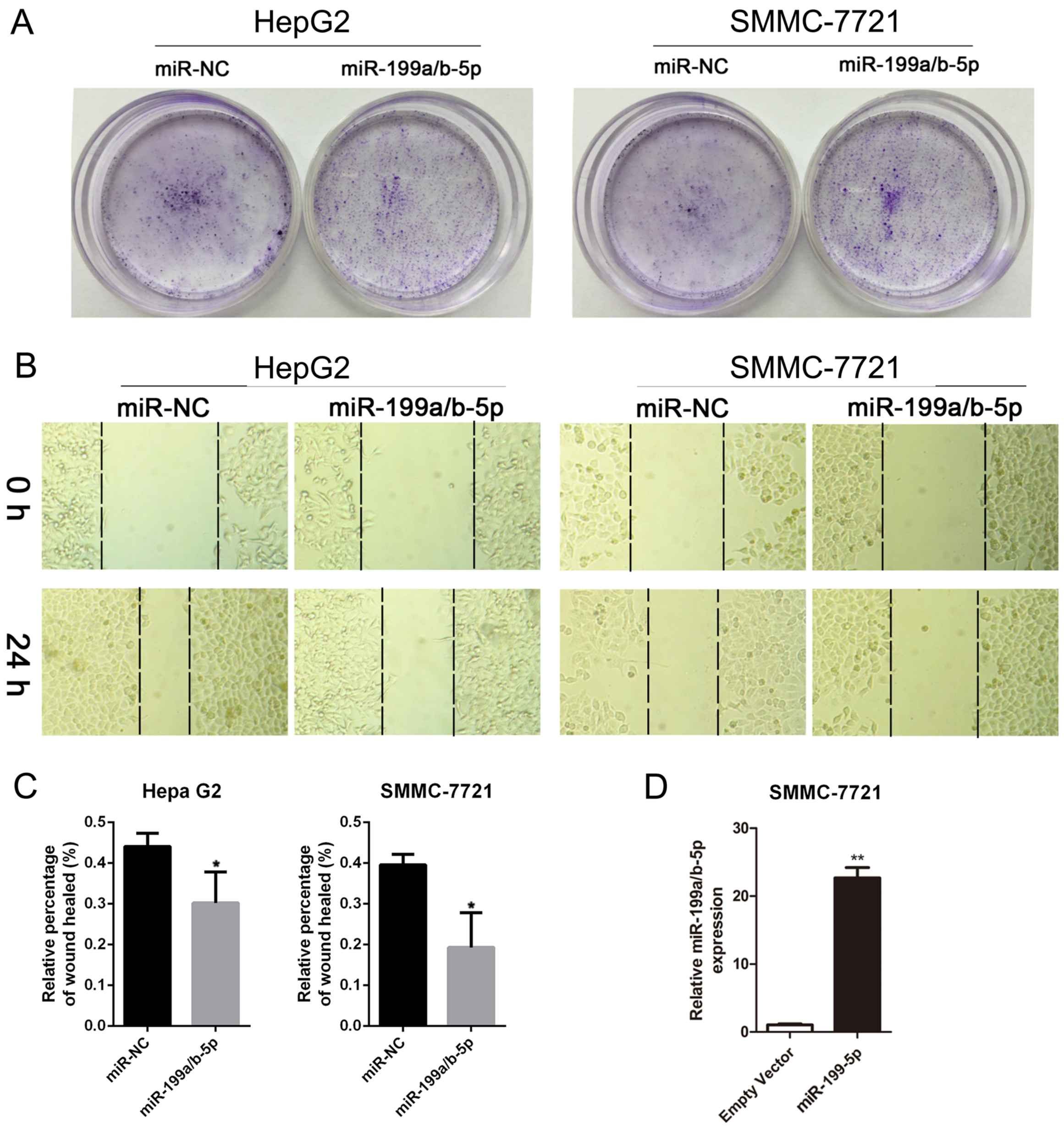

Supplementary Figure 2: Related to Figure 3. (A) Plate colony formation assays estimating cell proliferation in HepG2 and SMMC-7721 cell lines transfected with control or miR-199a/b-5p mimics. **(B, C)** Wound-healing assays measuring the cell motility of HepG2 and SMMC-7721 cell lines transfected with control or miR-199a/b-5p mimics. **(D)** qRT-PCR analysis of miR-199a/b-5p mRNA levels after miR-199-5p stable overexpression in SMMC-7721. The results were expressed as mean ± SD (*p<0.05, **p<0.01).

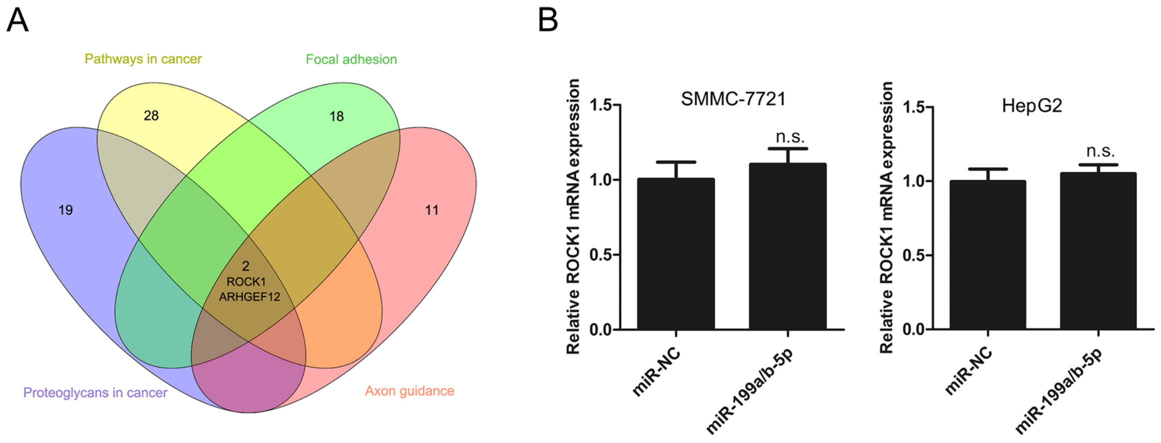

Supplementary Figure 3: Related to Figure 4. (A) ROCK1 and ARHGEF12 were found in the 4 most enriched cell signaling pathways by KEGG pathway enrichment analysis. **(B)** qRT-PCR analysis of ROCK1 mRNA levelsafter transfection with miR-199a/b-5p and miR-NC mimics in SMMC-7721 and HepG2 cells. The results were expressed as mean ± SD (*p<0.05, **p<0.01).

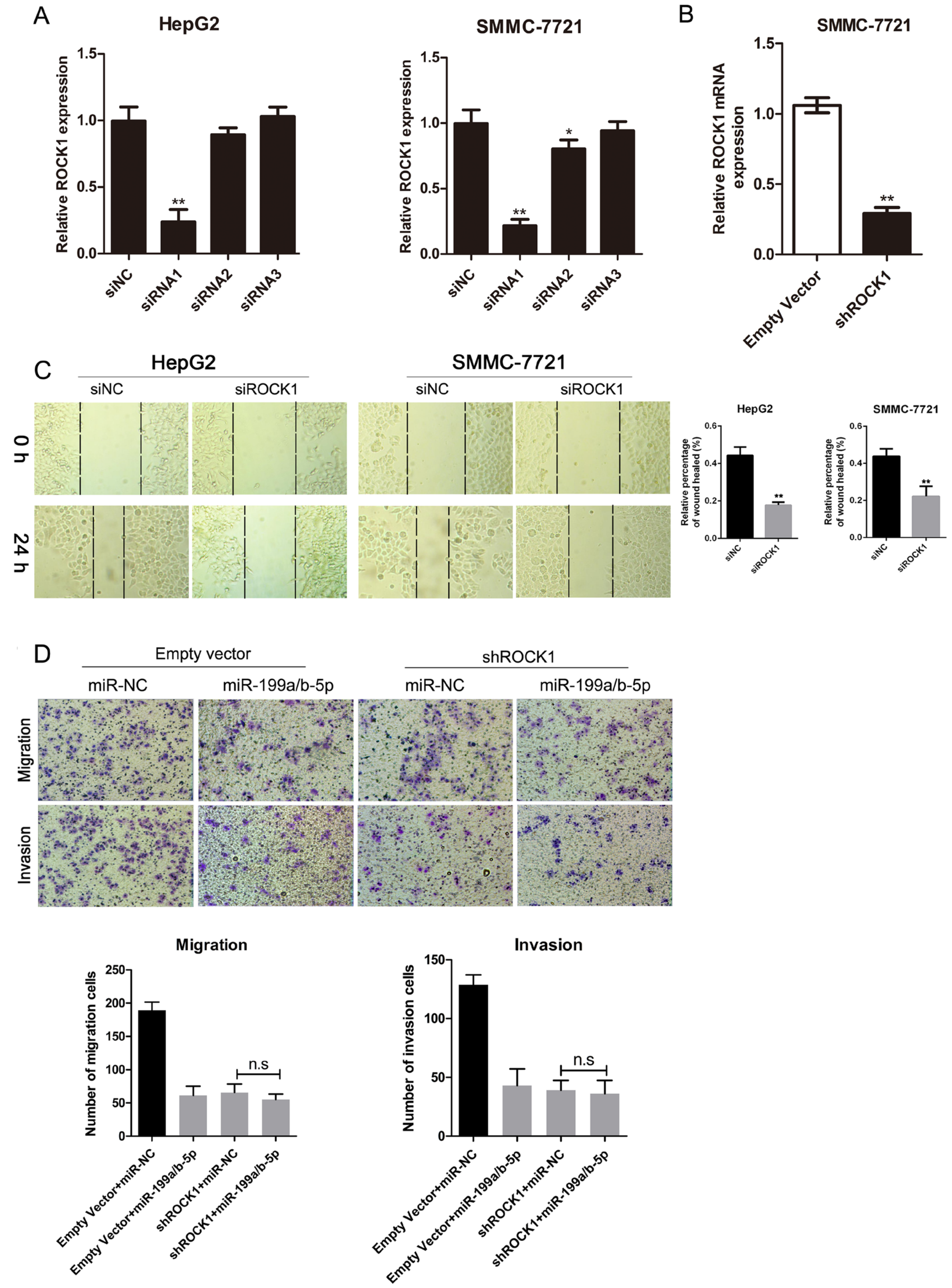

Supplementary Figure 4: Related to Figure 4. (A) qRT-PCR analysis of ROCK1 mRNA levelsafter transfection with three different siRNAs and siNC in HepG2 and SMMC-7721 cells. **(B)** qRT-PCR analysis of ROCK1 mRNA levels after transfection with empty vector and shROCK1 in SMMC-7721 cells. **(C)** Wound-healing assays measuring the cell motility of HepG2 and SMMC-7721 cell lines transfected with siNC or siROCK1. **(D)** Transwell assays determining cell migration and invasion potential of ROCK1 knockdown SMMC-7721 cells and control cells transfected with miR-NC or miR-199a/b-5p. The results were expressed as mean ± SD (*p<0.05, **p<0.01, n.s means no significance).

Supplementary Table 1: Related to Figure 1A, up/down-regulated miRNAs in HCC and matched normal liver tissues

miRNA	Average reads in normal liver	Average reads in HCC samples	$Log_2(T/N)$	LogCPM	P value	FDR
miR-199a-2	15,049	2,381	-2.0698204	12.41773769	4.05E-06	6.86E-06
miR-199b	17,749	2,802	-2.0684283	12.66089174	4.43E-06	7.20E-06
miR-199a-1	9,099	1,468	-2.0519113	11.69227063	1.51E-06	2.80E-06
miR-139	3,100	415	-2.0484013	10.15196109	2.46E-10	1.20E-09
miR-424	4,708	866	-2.0269011	8.804521073	2.04E-12	4.75E-10
miR-142	20,581	4,366	-1.8740857	10.98989967	2.16E-09	1.19E-07
miR-150	3,914	813	-1.6809660	10.62874728	1.36E-07	3.35E-07
miR-130a	850	150	-1.6777246	8.358254581	7.70E-10	3.34E-09
miR-451	14,160	2,120	-1.6745600	12.19902774	4.54E-05	7.08E-05
miR-145	15,077	3,895	-1.4136317	10.48896715	1.02E-05	0.000197
miR-486	2,162	469	-1.3137192	7.460127489	1.47E-05	0.00027
miR-10a	217,097	67,510	-1.1865342	14.46930259	0.001433	0.012487
miR-146b	8,415	13,722	1.3343589	13.20244	0.003382206	0.004122
miR-425	667	1,058	1.4746675	9.590966	1.06E-08	3.43E-08
miR-210	1,717	2,752	1.5007599	10.98051	0.000748986	0.001037
miR-30d	47,370	68,051	1.5159854	15.76871	2.21E-06	3.92E-06
miR-320a	1,912	3,355	1.6095479	11.1841	5.77E-07	1.13E-06
miR-25	34,645	55,384	1.6127962	15.34372	6.85E-09	2.43E-08
miR-338	1,572	3,291	1.6660130	10.95378	0.00016511	0.000248
miR-151	13,406	22,558	1.7731376	14.15701	1.37E-07	3.35E-07
miR-500a	873	1,524	1.7780683	10.19433	6.04E-09	2.36E-08
miR-93	10,892	19,411	1.7830911	13.83593	1.50E-07	3.45E-07
miR-21	492,611	1,006,951	1.9505564	19.52629	1.37E-08	4.12E-08
miR-452	423	1,677	2.6688992	9.963142	1.22E-10	6.81E-10
miR-217	913	13,125	4.3508002	12.46674	1.19E-10	6.81E-10
miR-182	6,424	89,884	4.5038293	15.53287	3.09E-17	5.66E-16
miR-10b	12,656	188,862	4.7526090	16.77752	4.35E-17	5.66E-16
miR-183	1,752	40,683	5.2025714	14.36661	2.70E-19	1.05E-17
miR-9-1	331	7,674	6.0367556	12.6403	9.81E-13	7.65E-12
miR-10b	12,656	188,862	4.7526090	16.77752	4.35E-17	5.66E-16
miR-9-2	326	7,626	4.620394096	9.239508317	8.59E-11	8.98E-09

Supplementary Table 2: Related to Fig4, 621 putative targets of miR-199-5p predicted by Targetscan

See Supplementary File 1

Supplementary Table 3: Related to Figure 4, enrichment of analysis of predicted miR-199a/b-3p targets in KEGG cell signaling pathway database

Pathway	Counts	p value
Proteoglycans in cancer	19	1.12E-05
Pathways in cancer	28	1.21E-05
Focal adhesion	18	6.05E-05
Axon guidance	11	0.002919
Hippo signaling pathway	12	0.003362
Protein processing in endoplasmic reticulum	12	0.007813
TGF-beta signaling pathway	8	0.009023
Circadian rhythm	5	0.010471
Endocytosis	15	0.013531
Arrhythmogenic right ventricular cardiomyopathy (ARVC)	7	0.014306
Chronic myeloid leukemia	7	0.015254
Signaling pathways regulating pluripotency of stem cells	10	0.016711
Basal cell carcinoma	6	0.018516
MAPK signaling pathway	14	0.026912
TNF signaling pathway	8	0.029111
Small cell lung cancer	7	0.031743
Regulation of actin cytoskeleton	12	0.034896
ErbB signaling pathway	7	0.035025
Renal cell carcinoma	6	0.035192
Pancreatic cancer	6	0.035192

Only the most enriched 20 cell signaling pathways of predicted miR-199a/b-3p targets in KEGG database were shown. A hypergeometic test was used to validate the significance with p value shown as indicated.
